# Mycobacterium-Infected Dendritic Cells Disseminate Granulomatous Inflammation

**DOI:** 10.1038/srep15248

**Published:** 2015-10-30

**Authors:** Jeffrey S. Harding, Aditya Rayasam, Heidi A. Schreiber, Zsuzsanna Fabry, Matyas Sandor

**Affiliations:** 1 Department of Pathology and Laboratory Medicine, School of Medicine and Public Health, University of Wisconsin; 2 Cellular and Molecular Pathology Training Program, University of Wisconsin Madison; 3 Neuroscience Training Program, University of Wisconsin Madison.

## Abstract

The disappearance and reformation of granulomas during tuberculosis has been described using PET/CT/X-ray in both human clinical settings and animal models, but the mechanisms of granuloma reformation during active disease remains unclear. Granulomas can recruit inflammatory dendritic cells (iDCs) that can regulate local T-cell responses and can carry bacteria into the lymph nodes, which is crucial for generating systemic T-cell responses against mycobacteria. Here, we report that a subset of mycobacterium-infected iDCs are associated with bacteria-specific T-cells in infected tissue, outside the granuloma, and that this results in the formation of new and/or larger multi-focal lesions. Mycobacterium-infected iDCs express less CCR7 and migrate less efficiently compared to the non-infected iDCs, which may support T-cell capture in granulomatous tissue. Capture may reduce antigen availability in the lymph node, thereby decreasing systemic priming, resulting in a possible regulatory loop between systemic T-cell responses and granuloma reformation. T-cell/infected iDCs clusters outside the granuloma can be detected during the acute and chronic phase of BCG and Mtb infection. Our studies suggest a direct role for inflammatory dendritic cells in the dissemination of granulomatous inflammation.

Infection with mycobacteria, including mycobacterium tuberculosis, results in the formation of granulomas. Granulomas are collections of mostly innate and adaptive immune cells organized around bacilli with a defined spatial arrangement and cellular composition^1^,^2^. They are necessary for protection, but are also inducers of tissue pathology^3^. These sites are the ecosystem that define the host-pathogen interface and are the space where bacilli are either eliminated or allowed to persist. The initiation of granuloma formation has been relatively well-described and studied. Activation of pattern recognition receptors (PRRs) on monocytes by mycobacterial lipids induces NFkB activation and TNFα release^4^,^5^. The activity of TNFα induces a cytokine storm, which supports the release of chemokines that recruit blood-borne monocytes, T-cells, B-cells, fibroblasts, and other cells^6^. Granulomas are highly dynamic sites, both in the activity of intracellular anti-microbial responses, as well as the continuous cell recruitment from the periphery needed to repopulate granuloma effector cells. We have previously shown that nearly 30% of mycobacterial granuloma dendritic cells are replaced in granuloma transplants after one week, and that the kinetics of this repopulation is different in early and chronic lesions, as well as cell-type dependent^7^,^8^,^9^.

One observation reported in animal models of tuberculosis using PET and CT imaging is the dynamism of granuloma appearance, disappearance, growth, and spreading during ongoing infection^10^,^11^,^12^. Even after bacterial infection and the subsequent burst of granuloma formation, lesions can disappear, while at the same time new ones can appear in previously non-granulomatous areas of the tissue. The disappearance of individual lesions has been described, and it is known that bacterial killing (with or without antibiotics) coincides with resolution of inflammation—lesions can also become fibrotic and/or calcified^13^. However, the mechanism that drives new lesion formation after acute infection is already established is not understood, despite the fact that the growth, spreading, and appearance of new lesions is a well-described clinical feature in tuberculosis patients with ongoing infection.

Here, we present data showing that dendritic cells (DCs) leave mycobacterial granulomas with bacteria. We show that mycobacterial-specific T-cells form contacts with emigrating DCs and induce the spreading of granulomatous inflammation in infected tissue. Inflammatory DC migration from granulomas may be key for the long-term, continuous renewal and chronic maintenance of granulomatous lesions.

## Results

### CD11c+ inflammatory dendritic cells are recruited to mycobacterial granulomas and get infected with BCG

**Figure 1:**
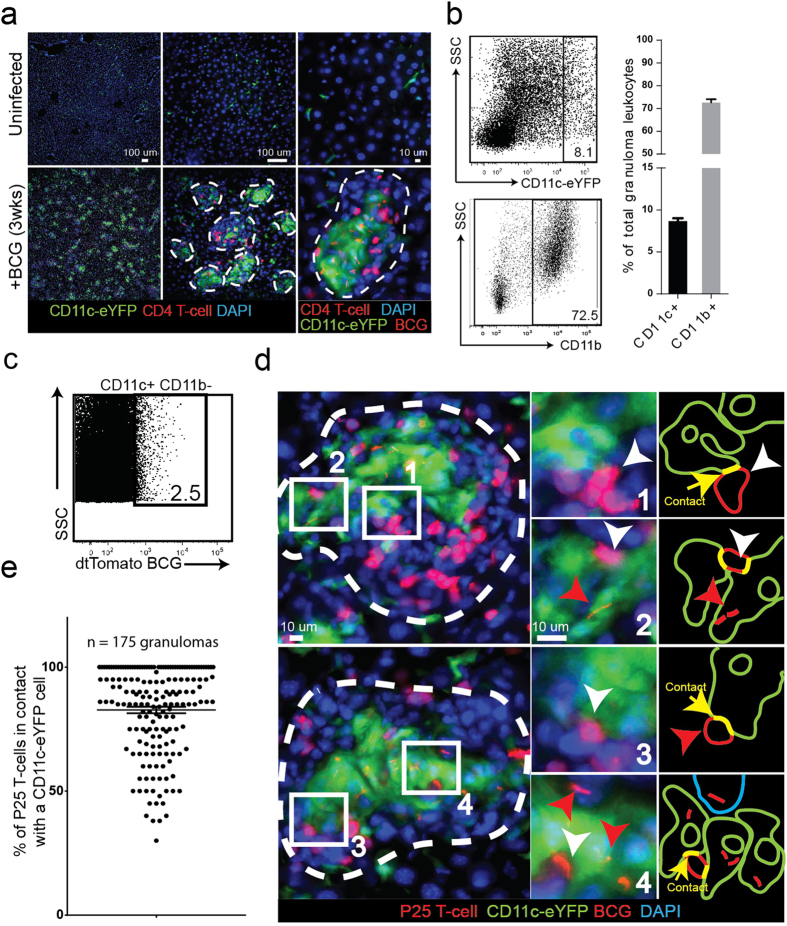
BCG-containing CD11c+ inflammatory dendritic cells in mycobacterial granulomas. CD11c-eYFP reporter mice (YFP expression driven by the CD11c promoter) were IP infected with tdTomato-expressing BCG and harvested 3 weeks later during acute infection. BCG-specific DsRed P25 T-cells were transferred into infected mice one week before harvest. (**A**) 50 μm-thick liver sections from uninfected and infected mice showing the accumulation of BCG-containing granulomas populated by CD11c+ and P25 T-cells. Individual granulomas outlined in white dotted line. (**B**) Flow cytometry plots of liver-isolated granuloma cells showing the proportion of CD11c+ and CD11b+ cells in the granuloma. Bar graphs shows average value among n = 13 mice among 3 replicate experiments. (**C**) Granuloma cells were isolated from Wt mice infected with tdTomato BCG and stained with CD11c+ antibodies. Flow plot shows the proportion of BCG-infected CD11c+ cells (colocalization of CD11c and tdTomato signal). Plot is representative of the average value (2.6%  +/− 0.2% SEM) calculated from n = 5 mice among 2 replicate experiments. (**D**) Both uninfected (panels 1 and 3) and BCG-infected CD11c+ cells (panels 2 and 4) form apparent contact with P25 T-cells in the same granuloma. Representative granulomas outlined with white dotted lines. White arrows indicate P25 T-cells, red arrows indicate BCG bacilli, green arrows indicate CD11c-eYFP cells, yellow arrows indicate contact between CD11c+ and P25 cell. The two granulomas shown in (**D**) were chosen as representative from an analysis of 175 total granulomas, plot shown in (**E**) (n = 7 mice among two replicate experiments. 25 granulomas per mouse distributed across five different 200x magnification fields). Micrographs in (**D**) come from 50 μm-thick tissue sections, and the entire depth of the tissue was scanned in order to verify that the CD11c+ cells identified as uninfected contained no BCG bacilli.

Most of the experiments described in this investigation take advantage of the dendritic cell reporter mouse strain (CD11c-eYFP), where the eYFP protein is expressed by the CD11c promoter. We first IP infected CD11c-eYFP mice with a high dose (1 × 10^7^ CFU) of a Bacillus Calmette-Guerin (BCG) strain of mycobacteria that was transfected with the plasmid encoding the tdTomato fluorescent protein. Acute infection develops within 3 weeks and results in the formation of bacilli-containing granulomas supported by massive recruitment of CD11c+ cells and other leukocytes, such as CD4+ T-cells, to the liver (Fig. 1a). CD11c+ cells are distributed both at the periphery and center of granulomas. Granulomas that develop during acute infection consist of a diverse leukocyte population, which includes 70% CD11b+ and 8–10% CD11c^high^ cells (Fig. 1b). Approximately 2% of the CD11c^high^ granuloma cells are infected with BCG, identified by colocalization of eYFP and tdTomato fluorescent signals (Fig. 1c). To investigate the relationship between CD11c+ and antigen-specific T-cells in the granuloma, we adoptively transferred 5 × 10^5^ DsRed-expressing P25 T-cells into BCG-infected mice 7 days before harvest. P25 T-cells, isolated from the P25 anti-85b^(240–254)^ mouse strain, are specific for a 14 amino acid peptide sequence of the BCG and Mtb-secreted 85b protein. While P25-expressed DsRed protein has a similar emission spectrum to tdTomato, these cells can easily be distinguished from bacterial rods by their morphology and size. BCG are also intracellular bacteria that reside in granuloma monocytes. After transfer, P25 T-cells proliferate in the lymph nodes and eventually migrate to, and populate, BCG-containing granulomas in the liver (Fig. 1d). In the granulomas, P25 T-cells can be found in contact with both uninfected (Fig. 1d, panels 1 and 3) and BCG-infected CD11c+ cells (Fig. 1d, panels 2 and 4). A random sampling of 175 granulomas selected from 7 mice showed that approximately 80% of granuloma-contained P25 T-cells were found in contact with CD11c+ cells (Fig. 1e). The association between antigen-specific T-cells and CD11c+ cells is a well-described feature of mycobacterial granulomas^14^. Dendritic cells are specialized for migrating to the lymph nodes with bacterial antigen, but during infection, CD11c+ inflammatory dendritic cells (iDCs) recruited from the blood can also support and stimulate IFNγ release from antigen-specific T-cells in the granuloma^14^. In summary, these experiments show that CD11c+ cells are recruited to BCG granulomas, a subset of which get infected and form apparent contacts with mycobacterial-specific T-cells.

### Contact between CD11c+ cells but limited antigen transfer in granulomas

Antigen-containing CD11c+ cells migrate to the lymph nodes and can transfer their antigen to lymph node-resident dendritic cells that are specialized for priming T-cells. We have previously shown the importance of this antigen transfer during BCG infection^7^, and it has also been described in Mtb infection^15^. To study the contact between CD11c+ cells, we utilized a model of granuloma transplantation, where BCG-infected liver from CD11c-eYFP animals was transplanted underneath the kidney capsule of uninfected and syngenic wild-type (Wt) mice^9^. Mice were harvested 7 days later, by which time CD11c-containing granulomas could be identified in transplanted tissue (Supplemental Fig. 1a, top micrographs). Fitting with previous reports of DC-DC transfer in the lymph nodes, we identified donor-derived CD11c+ cells in contact with recipient CD11c+ cells in the kidney-draining lymph node (rLN) of recipient mice (Supplemental Fig. 1a, bottom micrographs). Transplantation of BCG-infected liver from RAG KO mice into Wt mice demonstrated the increased dissemination of mycobacteria in the absence of adaptive immunity (Supplemental Fig. 1b). BCG from RAG KO donors were found in the rLN of Wt recipients and every bacteria was found inside a CD11c+ cell. We also used granuloma transplantation to describe DC-DC interactions in the granuloma. We transplanted BCG-infected liver from Wt mice into uninfected CD11c-eYFP recipients and harvested 7 days later (Supplemental Fig. 1c, top row). CD11c+ cells from the recipient migrate into transplanted granulomas (Supplemental Fig. 1c, second row), some of which get infected with BCG (Supplemental Fig. 1c, third and fourth row). We also found that some immigrating CD11c+ cells from the recipients closely line up with donor derived CD11c+ cells in transplanted granulomas (Supplemental Fig. 1c, bottom row).

Since we previously observed uninfected DCs in contact with P25 T-cells (Fig. 1d), we hypothesized that DC-DC interactions in the granuloma might result in antigen transfer from infected DCs to uninfected ones. It has previously been reported that interactions between immigrating DCs and local DCs in the lymph nodes are crucial for optimal anti-bacterial T-cell responses during some infections^15^. We isolated granuloma cells from mice infected with DsRed BCG and purified both uninfected and infected CD11c^high^ cells (by flow cytometry cell sorting) based on colocalization of CD11c-eYFP^high^ and BCG fluorescence. CD11c^high^ cells from to granulomas were sorted into BCG- and BCG+ fractions (Supplemental Fig. 2a). To preserve all of the few cells recovered from the CD11c^high^ BCG+ sort for the proceeding assay, we did not perform a post-sort run. However, during the actual sort, we noted the real-time readout of the instrument which indicated that the gate selected for CD11c^high^ BCG+ cells had greater than 99% purity. CD11c^high^ BCG- and CD11c^high^ BCG+ fractions were then incubated with cells from secondary lymphoid organs of P25 transgenic mice, and the activation of P25 CD4+ T-cells (CD4+ Vβ11+) in co-cultures was measured 24 hours later by expression of CD69 (Supplemental Fig. 2a,b). As expected, P25 T-cells were not activated when incubated with media alone (no CD11c^high^ cells) and were highly activated by the direct addition of 85b peptide, which is the mycobacterial protein that contains the P25 antigen. P25 T-cells were not activated when incubated with sorted uninfected CD11c+ granuloma cells (CD11c^high^ BCG- cells). However, a fraction of P25 T-cells were activated when incubated with sorted, granuloma-derived and infected CD11c+ (CD11c^high^ BCG+) cells (Supplemental Fig. 2b, lower row, and 2c). Though we hypothesized that antigen exchange between granuloma DCs could result in a P25-activating population of uninfected DCs, the results from these experiments show that this kind of antigen exchange is either non-existent or limited. It is also possible that uninfected DCs are not good activators of mycobacterial-specific T-cells regardless of the amount of antigen received from another DC for some other reason. Ultimately these data support the idea that uninfected DCs have limited access to 85b antigen, even though the 85b protein is a major secreted antigen.

### BCG-containing CD11c+ cells are outside the granuloma and in contact with P25 T-cells after the development of acute infection

**Figure 2:**
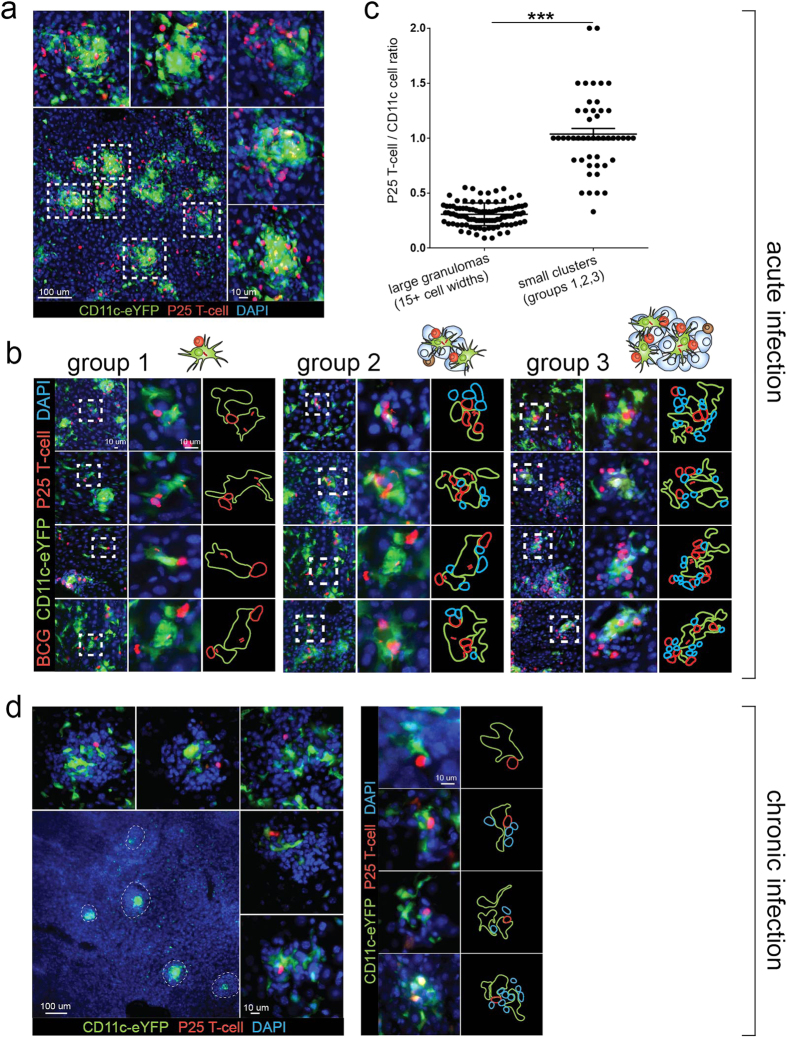
BCG-containing cells found outside the granuloma in contact with P25 T-cells during acute infection. CD11c-eYFP mice were IP infected with tdTomato BCG and 3 weeks (acute infection, (**A–C**)) or 10 weeks (chronic infection, (**D**)) later adoptively transferred (by IV injection) with 5 × 10^5^ DsRed P25 T-cells. Mice were harvested 7 days after cell transfer. (**A**) Representative low-magnification micrograph from 50 μm-thick sections of acutely-infected liver containing CD11c+ and P25 T-cells (bottom left image). Five representative granulomas (white dotted boxes, magnified in peripheral images) show the morphology and size (15–30 cells in diameter) of mature granulomas during acute infection. (**B**) Representative micrographs of BCG-containing, CD11c+ cells found outside mature granulomas and in contact with P25 T-cells. Clusters containing 1–4 cells (group 1, left panels), 5–10 cells (group 2, middle panels) and 11–20 cells (group 3, right panels) were identified. Images were taken from 50 μm-thick liver sections. (**C**) Comparison between of P25 T-cell to CD11c+ cell ratio in mature granulomas (15+ cells in diameter) and small cell clusters (groups 1,2,3), showing the unique cellular composition of each type of aggregate. 100 mature granulomas and 50 small cell clusters were randomly selected among n = 6 mice from 2 replicate experiments. The # of CD11c+ and P25 T-cells in each aggregate was counted using image J. Error bars are mean +/− s.e.m. student’s t-test used to determine statistical significance. ***P < 0.001. (**D**) Representative low-magnification micrographs from 50 μm-thick sections of chronically-infected liver containing CD11c+ and P25 T-cells (bottom left image, granulomas in white dotted circles). Higher magnification of 5 representative chronic granulomas shown in peripheral images.

During BCG infection, the overwhelming majority of CD11c+ cells recruited to the tissue are in granulomas (Fig. 1a). When DsRed P25 T-cells are transferred into infected mice before harvest, many CD11c+ cells are in contact with or adjacent to the transferred cells in granulomas (Fig. 1d). Since DCs have the inherent capacity to leave granulomas and migrate to the lymph nodes, and as most bacteria are contained in the granuloma, we asked if infected CD11c+ cells could be found outside the granuloma, and if they can be found in contact with P25 T-cells. To address this question, we IP infected CD11c-eYFP mice with tdTomato BCG and three weeks after infection (when granulomas have already formed in liver) adoptively transferred 5 × 10^5^ P25 T-cells. Tissue was examined 7 days later. By the time of harvest transferred P25 T-cells had proliferated, migrated to the liver, and populated BCG-containing granulomas. At this time, most of the granulomas are approximately 15–40 cell widths in diameter (Fig. 2a), and 10+ bacilli are found in a single 50 μm thick tissue cut. Mature granulomas consist mostly of monocytes, but also 8–10% CD11c+ cells (Fig. 1b). We identified a small number of CD11c+ cells infected with BCG that were not in the granulomas, but in contact with P25 T-cells (Fig. 2c, group 1). These extra-granuloma CD11c+ cells were also MHC II (IA^b^) positive (Supplemental Fig. 3). These DCs may represent the population in route from the granuloma to the lymphatic vessels that collect in the lymph nodes. We found single CD11c-P25 T-cells contacts, but also clusters that contained several CD11c+ and P25 T-cells. (Fig. 2b, group 2,3). Larger clusters containing 2 or more CD11c+ cells also contained other leukocytes that were neither CD11c-eYFP cells nor P25 T-cells (Fig. 2b, blue outlines). Small cell clusters (groups 1,2,3) also had a characteristic ratio of CD11c+ to P25 T-cells that was nearly 3-fold higher than the ratio measured in mature granulomas (Fig. 2c). In mice, IP infection with BCG starts resolving after 3 weeks, which involves a steady decline in the number of granulomas and bacilli. We identified extra-granulomatous DC/P25-cell clusters in chronically-infected mice (10 weeks after infection) that were histologically similar to those identified in acutely-infected mice. One week after adoptive transfer, P25 T-cells had already been recruited to granulomas, though in lower number than acute granulomas (Fig. 2d, left images). We identified both individual CD11c-P25 T-cell contacts, as well as larger clusters that contained more than one CD11c+ cell (Fig. 2d, right images). Interestingly, chronic BCG infection in mice results in granulomas with many similarities to those in asymptomatic Mtb-infected patients, including a many-fold reduction in the number of bacilli, a decrease in cell-mediated anti-microbial responses, and a persistent population of bacilli in chronic granulomas^16^.

**Figure 3:**
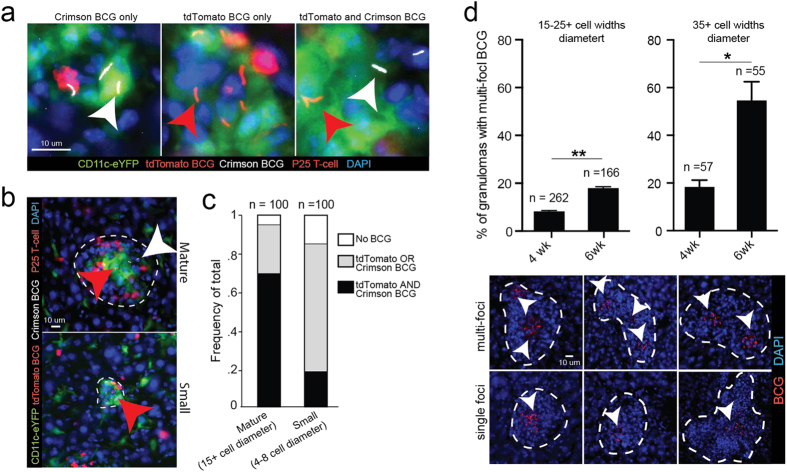
Small DC/P25 T-cell clusters more likely to contain a single BCG strain in mice infected with multiple subtrains. (**A–C)** CD11c-eYFP mice were IP infected with a 1:1 (1 × 10^7^ total CFU) mixture of tdTomato BCG and Crimson BCG and 3 weeks later (adoptively transferred (by IV injection) with 5 × 10^5^ DsRed P25 T-cells. Representative areas inside granulomas that contain tdTomato BCG (red arrows) only, Crimson BCG (white arrows) only, or both, in 50 μm-thick liver sections. Low magnification micrographs of the entire granuloma from images in (**A**) are outlined in white dotted lines in (**B**) and show two representative sizes of granulomas. (**C**) Quantification of the frequency of granulomas that contain no BCG, tdTomato or Crimson BCG only, or tdTomato and crimson BCG in 100 mature (15+ cell diameter) and small (4–8 cell diameter) granulomas randomly selected from n = 6 mice. (**D**) Frequency of granulomas containing multi-focal BCG in 50 μm-thick liver sections in mice 4 or 6 weeks after IP infection with 1 × 10^7^ CFU tdTomato BCG. Representative single and multi-focal granulomas outlined in white dotted lines. White arrows identify individual BCG foci. Granulomas were randomly selected from n = 4 mice in a single experiment. Error bars are mean +/− s.e.m. Student’s t-test used to determine statistical significance. *P < 0.05; **P < 0.01. Number of total granulomas measured for each group shown above individual column.

We hypothesized that new granulomas could be forming from emigrating BCG-infected CD11c+ cells. To investigate this possibility, we IP infected mice with an equal 1:1 mixture (1 × 10^7^ total CFU) of two different recombinant BCG populations that expressed either the Crimson or tdTomato fluorophore, so that each BCG population could be traced independently. Both transfected populations were derived from the same founder BCG culture. If new lesions form from granuloma-emigrating CD11c+ cells, which if infected most likely contain only one kind of bacilli, then new granulomas are more likely to be populated by only a single fluorescent strain that are progeny of the first founder bacilli. Furthermore, given that new lesions are more likely to be smaller and contain fewer cells, we hypothesized that there should be a correlation between granuloma size and the probability of containing only a single fluorescent strain. We divided granuloma size into two size categories, 15+ cell widths (“mature”) and 4–10 cells (small), and counted the proportion of granulomas within each category that contained no BCG, tdTomato or crimson BCG or tdTomato and Crimson BCG (Fig. 3a,b). We found marked differences in the proportions of each size category that contained 1 or 2 strains of BCG. Nearly 95% of all mature granulomas (15+ cells widths) contained both Crimson and tdTomato BCG, while only 20% of small granulomas (4–10 cell widths) contained both strains (Fig. 3c). Small lesions where therefore approximately 5 times more likely to contain only a single strain of BCG compared to larger lesions. In addition to forming new granulomas, we also hypothesized that emigrating, BCG-infected DCs might be able to spread the size and/or locations of BCG containment within an existing granulomas. To test this hypothesis, we IP infected mice with tdTomato BCG and measured the difference in frequency of multi-focal lesions (granulomas that contain concentrated and distinct foci of BCG bacilli) 4 and 6 weeks later. Among both large (15–25 cell widths) and extra-large (35+ cell widths) granulomas, we measured a nearly 2.5 fold increase in the frequency of multi-focal lesions at 6 weeks post infection compared to 4 weeks (Fig. 3d). These data suggest that BCG can spread within existing granulomas. Taken together, these experiments support the idea that mycobacterium-infected DCs may disseminate and/or perpetuate granulomatous inflammation even after acute infection has already resulted in a population of mature granulomas

### Reduced migration of BCG-infected Dendritic Cells

**Figure 4:**
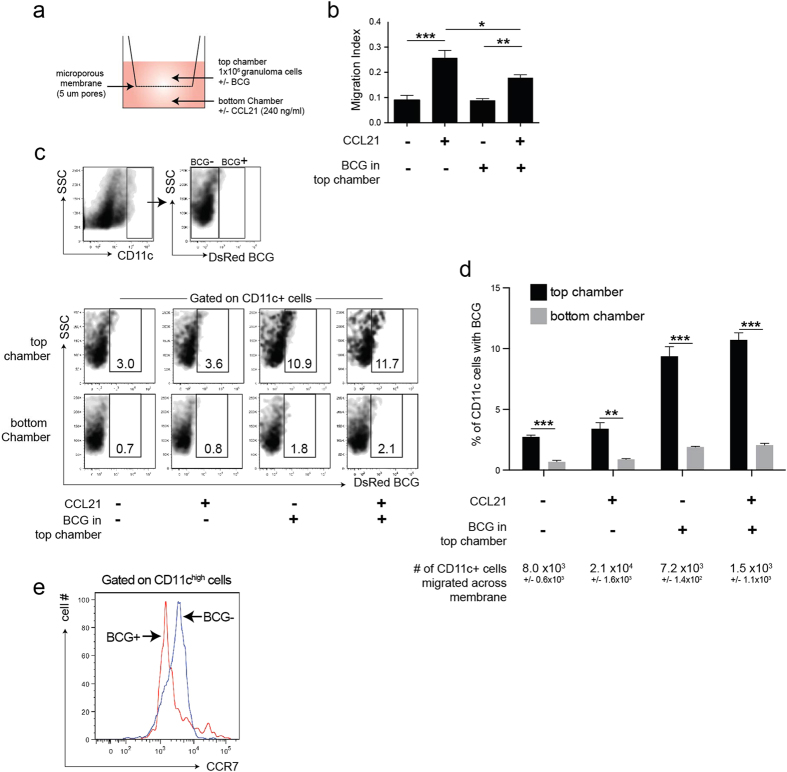
Decreased migration of BCG-infected dendritic cells. (**A**) Schema of migration assay. 1 × 10^6^ granuloma cells were loaded into the top chamber of a 5 μm microporous membrane and cells that migrated through into the bottom chamber after 24 hours were collected. Bottom chambers contained media alone or CCL21 (240 ng/ml), top chambers contained granuloma cells alone or granuloma cells+ 2 × 10^4^ BCG bacilli from fresh culture. (**B**) Migration index of CD11c+ cells, measured as the proportion of cells that migrated across the barrier and into the bottom chamber after 24 hours. (**C**) Top panels: Flow cytometry plots showing the gating and identification of uninfected, or BCG-infected CD11c+ cells. Bottom panels: representative flow cytometry plots showing the proportion of BCG-infected (CD11c+ DsRed+) cells in the top and bottom chambers after 24 hours. (**D**) Quantification of data from C. (**E**) CCR7 expression on CD11c+ DsRedBCG+ and CD11c+ DsRedBCG- cells isolated from acute BCG granulomas. The data show the preferential migration of uninfected CD11c+ cells across the barrier. Error bars mean +/− s.e.m. Student’s t-test (**D**) and One-way ANOVA (**B**) used to determine statistical significance. *P < 0.05; **P < 0.01; ***P < 0.001.

We next investigated if BCG infection of CD11c+ cells resulted in reduced migrational capacity of these cells. We measured the capacity of granuloma cells from mice infected with DsRed BCG to migrate across a microporous membrane (5 μm pore size) towards CCL21 (Fig. 4a), a chemokine that binds CCR7 and supports cell migration towards lymph nodes. We isolated granuloma leukocytes from murine liver 3 weeks after IP infection with DsRed BCG and loaded 1 × 10^6^ cells onto the top of the membrane. After 24 hours, the proportion of cells that migrated across the barrier (the migration index) was approximately 10%, or 1 × 10^5^ cells, when no CCL21 was added to the bottom chamber. There was a 2.5 fold increase in the migration index when the bottom chamber contained CCL21, but the increase was suppressed by nearly 30% when BCG (2 × 10^4^ BCG bacilli) were added directly to the granuloma cell-containing top compartment (Fig. 4b). The addition of BCG did not affect the migration index of granuloma cells in the absence of CCL21. We next asked if there was a difference in the migration between uninfected and infected CD11c+ granuloma cells by measuring the proportion of infected CD11c+ cells (colocalization of CD11c cell staining and DsRed BCG fluorescence) on the top (loaded) and bottom (migrated) cell populations. In all conditions measured, including the addition of CCL21 and/or extra BCG, the frequency of CD11c+ cells in the bottom chamber that also contained BCG was 4–6 fold less than the frequency measured among CD11c+ cells in the top chamber (Fig. 4c,d). While the addition of CCL21 to the bottom chamber increased the total number of migrated CD11c+ cells, it did not change the proportion of CD11c^high^BCG+ and CD11c^high^BCG- cells in each chamber. Although the addition of BCG to the top chamber increased proportion of infected CD11c+ cells in both the top and bottom chambers, there was still a 6-fold increase of CD11c^high^BCG- cells in the bottom chamber. One fact that could explain the differences is that CD11c^high^BCG+ granuloma cells also had reduced CCR7 expression compared to CD11c^high^BCG- ones (Fig. 4e), which is in agreement with a previous report describing reduced CCR7 expression on mycobacteria-infected DCs and reduced migration of infected DCs^17^. In summary, these data show that infected CD11c^high^ granuloma cells are less likely to migrate across a transmembrane barrier than uninfected CD11c^high^ granuloma cells, and that this correlates with reduced CCR7 expression.

### Infected DCs outside of Mtb-induced granulomas

**Figure 5:**
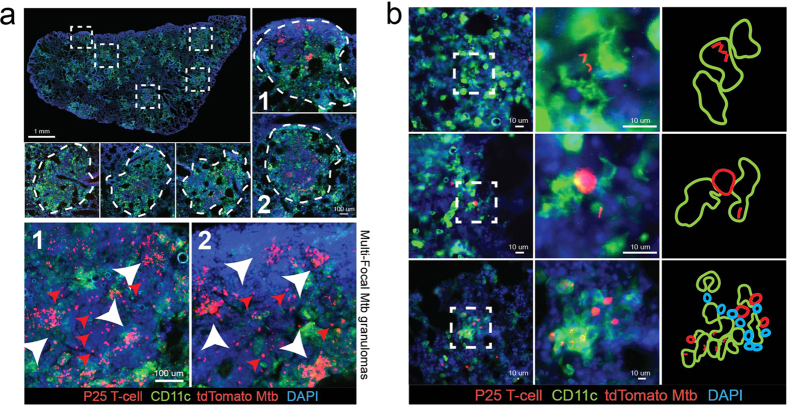
Infected CD11c+ cells found outside Mtb-induced granulomas. Wt mice were aerosol-infected with 500 CFU tdTomato-expressing mycobacterium tuberculosis (Mtb, strain H37Rv). 4 weeks after infection, 5 × 10^5^ DsRed-expressing P25 T-cells were transferred into infected animals, and then tissue harvested 10 days after transfer (**A**) Entire left lung (composite of 50 100x micrographs from 50 μm thick lung-sections) from Mtb-infected mouse. Representative granulomas selected from the composite image are outlined by white boxes and shown at higher magnification (white dotted lines). Two of the granulomas (“1” and “2”) containing multi-focal Mtb are shown at high magnification in the bottom two micrographs. White arrows point to separate Mtb foci within single granulomas. (**B**) Micrographs of Mtb-containing CD11c+ cells with distinct dendritic cell morphology found alone outside granulomas, or in small cell clusters containing DsRed P25 T-cells (transferred 10 days prior to harvest).

We next asked if CD11c+ cells could be found in contact with P25 T-cells outside the granulomas in the lungs of mice infected with virulent Mycobacterium tuberculosis (Mtb). We transfected the H37Rv strain of Mtb with the plasmid encoding the tdTomato fluorophore. We aerosol-infected CD11c-eYFP mice with 500 CFU tdTomato Mtb, adoptively transferred 5 × 10^5^ dsRed P25 T-cells 25 days later, and harvested tissue 10 days after transfer. Mtb lung granulomas are much larger than BCG liver granulomas, but they are similarly populated by CD11c+ cells (Fig. 5a, top images) as well as antigen specific P25 T-cells (Fig. 5a, bottom images, red arrows). Like BCG granulomas, during acute infection almost all Mtb bacilli are contained within granulomas in the lung. One of the distinguishing features of Mtb lung granulomas is that a subset of them contain multi-focal populations of bacilli (Fig. 5a, bottom images, white arrows). A single granuloma may contain two or more densely packed foci of Mtb, and so the total distribution of bacilli is non-homogenous. Multi-focal lesions have also been observed in Mtb-infected monkeys, and their appearance has been described not as a result of separate coalescing granulomas, but from “granuloma spreading” from a single inflammatory site^12^. We also identified extra-granulomatous Mtb, which were found almost exclusively inside CD11c+ cells (Fig. 5b, top two rows). Some Mtb-containing CD11c+ cells with dendritic cell-like morphology found outside granulomas were in contact with P25 T-cells (Fig. 5b, middle row). We also found small clusters of CD11c+ cells, Mtb, and P25 T-cells that were significantly smaller than typical granulomas and contained 10–30 total cells (Fig. 5b, bottom row). The data from these Mtb infection experiments support the possibility that Mtb-infected DCs emigrating away from lung granulomas come in contact with specific T-cells near the periphery of lesion, which could result in multi-focal structures and granuloma spreading.

## Discussion

Granuloma formation in the liver begins 2 weeks after IP infection with BCG, and by 3 weeks the tissue has the maximal bacterial load and number of mature lesions. Granulomas are the sites where almost all mycobacteria are sequestered. Mature granulomas that we isolate from the liver are constituted by 70% macrophages, approximately 5–10% CD4 T-cells, and 5–10% DCs (Fig. 1b). In the granuloma, inflammatory DCs (iDCs) are the dominant DC, which unlike tissue-resident DCs are recruited during inflammation and differentiate from blood-born monocytes^14^,^18^. In the acute granuloma, iDCs enhance the local release of IFNγ from T-cells^19^,^20^,^21^. Although some evidence shows that the enhancement is suboptimal due to low antigen availability^22^, the local re-boosting of T-cells is still critical for anti-microbial immunity in the granuloma. iDCs can tolerize T-cells just like dendritic cells of other lineages^23^ and cross-present to CD8 T-cells as well^11^,^24^,^25^,^26^,^27^. Importantly, they can also move from the site of inflammation and deliver antigen or inflammatory agents to the lymph nodes^28^,^29^,^30^,^31^,^32^, and CCR7 is considered an important regulator of this movement^28^,^31^,^33^.

Recent studies have investigated the importance of iDC/local DC interactions in the lymph node for T-cell priming. We and others have described the cooperation of migratory and lymph node DCs during mycobacterial infection^7^,^34^, but it has also been described in viral and fungal infections as well^30^,^35^. These studies establish that iDCs transport mycobacterial agents to the lymph nodes. Recently, Srivastava *et al*. described a mechanism where migratory DCs release undegraded mycobacterial protein, which gets picked up by lymph-node resident DCs, but does not involve transfer of whole bacterium and is distinct from apoptosis or exosome shedding^15^. These data also suggest that mycobacteria-infected migratory DCs are weak primers of the CD4 T-cells needed for protection during Mtb infection^36^,^37^,^38^,^39^, so transfer of antigen in the lymph nodes passes bacterial antigen to lymph node-resident DCs that are better T-cell activators. We thought that antigen transfer could support iDC function not only in the lymph node, but also in the granuloma. This was based on our data showing that host and donor-derived DCs lined up in granuloma transplants, that recipient transplant-infiltrating DCs from the recipient got infected, and that seemingly non-infected DCs in the granuloma can be found in contact with P25 T-cells. However, after separating infected and non-infected iDCs from granulomas, we demonstrated that if antigen transfer occurs, it is not enough to activate P25 T-cells. These findings suggest that in granulomas, unlike lymph-nodes, DC/DC antigen transfer is not efficient and that activation of T-cells in the lymph nodes likely occurs via DCs that contain antigen, but not full bacilli. They also support the previous suggestions that transfer of live bacteria in the lymph node is required for anti-bacterial immunity.

**Figure 6:**
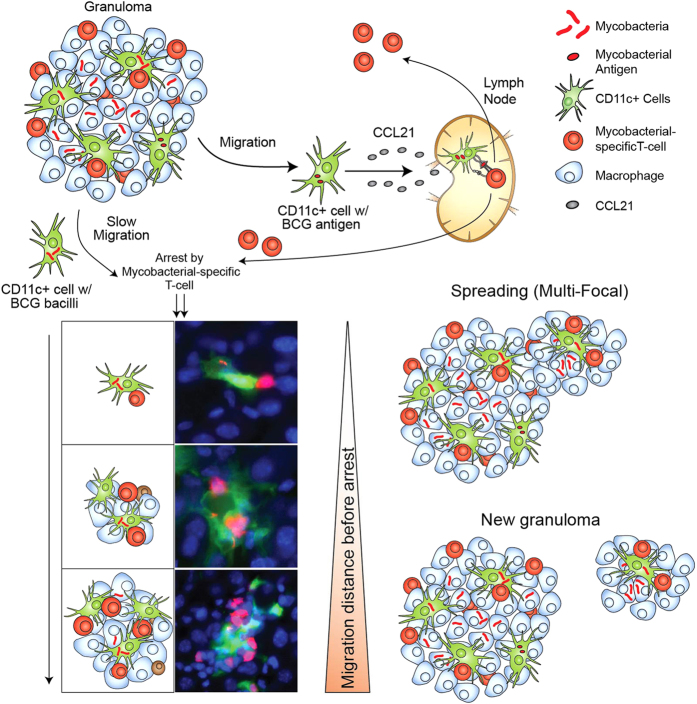
Dendritic cell-induced granulomatous renewal and spreading. Proposed model of granuloma spreading and reformation. CD11c+ cells emigrate from mycobacterial granulomas and a subset of them get infected. Some are able to reach the lymph node where they can prime mycobacterial-specific T-cells. Activated T-cells then migrate into the infected tissues towards granulomas. Granuloma-emigrating CD11c+ cells with bacteria have reduced migrational rates, which supports the likelihood of their contact with incoming and activated mycobacterial-specific T-cells. The site of contact then becomes a new foci for the accumulation of infiltrating monocytes and lymphocytes. The distance travelled by emigrating, BCG-containing CD11c+ cells dictates whether the site becomes a new granuloma, or simply enlarges the periphery of the existing granuloma.

Since inflammatory DCs carry mycobacteria from the granuloma to the lymph nodes^40^, we used CD11c-eYFP mice to look for infected CD11c+ cells outside mycobacterial granulomas. Many of those found were also coupled to P25 T-cells, indicating their capture by mycobacterial-specific T-cells (Figs 3 and 4). This arrest cannot be absolute since DCs have also been shown to be disseminators of bacteria to the lymph nodes^7^,^15^. In the absence of T-cells, we found many bacteria contained in DCs of lymph nodes, showing that these T-cells at least partly regulate infected DC dissemination out of the granuloma-containing sites. Our results show that only a portion of mycobacterial-containing DCs may reach the lymph node. Many are arrested *in situ*, and the distance traveled before arrest may dictate the inflammatory character of the new foci, whether it is the spreading of an existing lesion or the formation of a new one (Fig. 6). We have shown this process both in the liver (IP BCG infection) and the lung (Mtb aerosol infection), though it appears that new lesion formation is common in the liver while spreading is more common in the lung. The start of these lesions is enriched for mycobacterial-specific T-cells and DCs, but as cells accumulate around the initial DC-T-cell foci, the growing lesions take on the character of mature lesions, which contain mostly monocytes/macrophages. Using granuloma suspensions in-vitro, we showed that the migration of DCs can be driven by CCL21 and that migration is somewhat less efficient when DCs are infected (Fig. 4). Our model shows that iDCs, in addition to supporting granuloma T-cell function and bacterial dissemination, also seed granuloma reformation during ongoing granulomatous inflammation. This is fitting with a previous study by Lin *et al.*, who used specific SNP-identified strains of mycobacteria to show that most granulomas are initiated by single bacterium^12^. Our data suggests that this single bacterium may be contained within an emigrating dendritic cell. T-cell capture of infected dendritic cells in infected tissue can serve as a regulatory loop between local and systemic immunity. The capture diminishes antigen availability for T-cell priming in the lymph node and decreases the quantity of bacterial-specific T-cells. Lower availability of specific T-cells decreases the frequency of capture and promotes more antigen reaching the lymph node.

While our data show that DC-T-cell interactions may spread granulomatous inflammation *in situ*, they may also limit the spread of bacteria to peripheral sites. DCs capture mycobacteria in the granuloma, then process and present their antigens in the lymph nodes to activate adaptive B and T-cell responses. However, mycobacteria can prevent their own death and degradation by escaping the monocytoid phagosome, which essentially turn DCs into disseminators of live mycobacteria. Arrest of mycobacteria-containing DCs by antigen-specific T-cells *in situ* might therefore limit the spread of bacilli to extrapulmonary organs such as lymph nodes, spleen, pancreas, liver, bone marrow, and the central nervous system. The optimal balance between generating protective T-cells responses and preventing bacterial spread may require an optimal threshold of DC emigration vs. DC capture, which may ultimately be dictated by the availability of antigen-specific T-cells *in situ*.

We identified extra-granulomatous, mycobacteria-infected CD11c+ cells during both BCG liver and Mtb lung infection. Granulomas induced by virulent Mtb and avirulent BCG differ in many respects including size, caseation, and bacterial load. The organ-specificity of each granuloma, as well as different infectious doses, also influences how each is formed and maintained. For certain aspects of granuloma biology, there are limitations on what can be learned about one by studying the other. There are, however, many similarities, including the dominance of macrophages, requirement for IFNγ-producing CD4 T-cells, and abundance of TNFα, to name a few. Importantly, CD11c+ DCs are present in both lesions, and their role in transporting mycobacterial antigen and initiating adaptive immunity well-described^41^. Our observations of extra-granuloma, infected DCs in both BCG and Mtb infectious models suggest that granuloma spreading via DC migration and T-cell capture may be fundamental to granuloma biology across many granuloma-inducing pathogens and in many tissues. The character of this spreading (new lesions vs. expansion of existing lesions, for example) would likely be dictated by the character of the granuloma, the tissue it is in, and the pathogen that induced it.

Inflammatory lesions are formed by short-lived effector cells. Granulomas can be cleared through a number of mechanisms, including phagocytosis, calcification, and fibrosis. The reformation process explained here could provide an explanation for how granulomas contain mycobacteria in the long-term despite continual granuloma clearance, and also explain the clinical observation of new granuloma appearance during ongoing infection. Similar reformation could be common of other granuloma diseases as well. Our data shows that this process may be supported by infection-induced depression of dendritic-cell expressed CCR7 and reduced migration towards CCL21-dependent gradients. Granulomas are required to contain and prevent the spread of Mtb, and so reduced DC migration may be an evolutionary advantage to support renewal of containment sites by providing more time for specific T-cells to capture DCs in the tissues where they originate. Decreased movement of dendritic cells and suppression of important chemokine receptors during mycobacterial infection has been reported by others^17^,^19^,^42^. In summary, inflammatory DCs can disseminate granulomatous inflammation and understanding this process, along with the biology of DC movement, may help develop treatment for mycobacterial and other granulomatous diseases.

## Methods

### Mice

Wild type C57BL/6 and RAG KO (STOCK B6.129S7-Rag1tm1Mom/J) mice were purchased from Jackson laboratory (Bar Harbor, ME). CD11c-eYFP (STOCK B6.Cg-Tg (Itgax-EYFP)1Mnz/J)^43^ mice were originally from Michael C. Nussenzweig, and are now bred and maintained in-house. P25 Mice (STOCK C57BL/6-Tg(TcraTcrb)Ktk/J)^44^,^45^, which contain CD4+ cells that recognize the anti-85b^(240–254)^ epitope restricted to MHC class II IAb, were originally gifted by Alan Sher (NIH) and are also bred and maintained in-house. DsRed mice (STOCK Tg(CAG-DsRed*MST)1Nagy/J)^46^, were originally purchased from Jackson and now maintained in-house. The DsRed-expressing P25 T-cells used in the experiments from this report were isolated from the F1 generation of a cross between homozygous DsRed and homozygous P25 mice. All mouse strains, including RAG KO, CD11c-eYFP, P25, and DsRed, were on the C57BL/6 background, and restricted to the MHC class II IA^b^.

### Ethics Statement

Mice were housed and bred in microisolator cages in pathogen-free facilities at the University of Wisconsin Animal Care Unit (Madison, WI). All experimental procedures were performed in accordance with the guidelines of the Institutional Animal Care and Use Committee and all animal protocols were approved by the University of Wisconsin Institutional Animal Care and Use Committee.

### BCG and Mtb Infection

DsRed BCG was obtained from Dr. Lalita Ramakrishnan. Wild-type BCG (Pasteur Strain) was grown at 37 °C in Middlebrook 7H9 media (VWR) supplemented with 10% OADC and 0.05% Tween (Fisher), and then transfected with plasmids encoding either the tdTomato or Crimson protein. GFP-expressing plasmids were generously provided by Dr. Glen Fennelly (Albert Einstein University, NY). After transfection, BCG was again grown cultured in 7H9 media and then frozen at −80 °C. Prior to infection, frozen stocks were thawed and briefly sonicated. Mice were intraperitoneal (IP) injected with 1 × 10^7^ CFU BCG in 100 μL PBS. Infection via IP injection results in peak bacterial burden and number of granuloma-forming cells in the liver approximately 21 days after infection. For experiments involving infection with both tdTomato and Crimson BCG, mice were injected by IP injection 5 × 10^6^ CFU of each strain. Mtb strain H37Rv (ATCC) was transfected with the tdTomato plasmid, and grown at 37 °C in Middlebrook 7H9 media (VWR) supplemented with 10% OADC and 0.05% Tween 80 (Fisher). Live tdTomato H37Rv culture was taken during midlogarithmic phase and loaded directly into an Inhalation Exposure System (Glas-col). Mice were infected with approximately 500 CFU aerosolized bacilli using a pre-programmed nebulization protocol. Growth of Mtb, infection, and collection of tissue was done in a BSL3 facility under approved biosafety guidelines.

### Cell Isolation and Flow Cytometry

Isolation of liver granuloma cells was performed as previously described^47^. Briefly, infected livers from experimental groups were homogenized in a tissue blender, and granulomas were separated from hepatic cells based on size. In this separation, granuloma and aggregated cell clusters settle to the bottom of the tube more rapidly than single cells in the homogenate. After the entire homogenized liver was transferred to a 50 ml conical tube and allowed to settle for approximately 1 minute, the top 35 mL (containing mostly single cells) was discarded, and replaced with 35 mL of RPMI media. The entire tube was then inverted 3–5 times, and the process repeated once more. Isolates were then washed twice in RPMI and digested with 5 mg/mL type I collagenase (Sigma-cell Aldrich, St. Louis, MO) at 37 °C for 40 min while shaking. After digestion, removal of excess collagen was completed through repeated pipetting of tissue homogenate and filtering through a 70 μm nylon cell strainer (BD Bioscience, San Jose, CA). Liver granuloma isolates were then treated with red blood cell lysis buffer and washed twice with RPMI. 1 × 10^6^ cells were washed twice in FACS staining buffer (1% BSA in PBS) and incubated with fluorochrome-labled antibodies while on ice. Antibodies against CD11c (HL3), CD11b (Mac-1), and CCR7 (4B12) were purchased from BD Biosciences (San Jose, CA). Antibodies against MHC II IA^b^ (RUO) were purchased from eBioscience. All antibody-staining cocktails contained 40 μg/mL unlabeled 2.4G2 mAb to block non-specific Fc receptor binding. Data from stained cell isolates were collected on an LSRII (BD Biosciences) and analyzed with FlowJo v. 8.7 (TreeStar).

### Granuloma Transplantation

Granuloma transplantation was performed as previously described^7^. Briefly, mice were anesthetized with a ketamine/xylazine mixture, and given a subcutaneous injection of meloxicam for pain. After removal of hair and application of iodine, a 1-cm longitudinal incision through the skin and peritoneum was made on the dorsal side of the mouse toward the posterior end, above the last rib and hip joint. The kidney was withdrawn and a 1 mm long incision along the kidney capsule was made. The capsule was drawn away from the kidney with forceps and two pieces of BCG-infected donor liver approximately 25 mg (+/− 10%) in mass were inserted into the space underneath the capsule. The peritoneum was sutured and the skin incision close with surgical staples. Mice received kanamycin (5 mg/kg) in their water for several days starting 1 day prior to surgery (All BCG strains in this report contain plasmids encoding a kanomycin resistance gene). Seven days after transplantation, kidneys were removed and fixed for 24 hours in PBS solution containing 3% PFA and 25% sucrose. A longitudinal slice was made through the kidney at the transplant position. Both kidney halves were frozen in OCT and 10 μm slices were mounted onto slides before staining with CD11c-APC and DAPI.

### Immunohistochemistry

Sections from liver of BCG infected mice (left, median, and right lobes), kidneys and lymph nodes from transplant recipients, and the entire left lung of Mtb-infected mice, were fixed in PBS solution containing 3% PFA and 25% sucrose for 24 hours and frozen in O.C.T (Tissue-Tek Sakura, Torrance, CA). Tissue sections were cut and mounted onto slides, fixed in acetone for 10 min and washed with PBS. Sections were stained with CD11b, CD11c, or CD4 antibodies for 3 hours, washed with PBS and FACS, and then mounted with ProLong Gold Antifade with DAPI (Invitrogen, Carlsbad CA). Images were obtained with an Olympus BX41 Fluorescent microscope (Leeds Precision Instruments). All micrographs in which the analysis was based on identification of individual infected cells, cell-cell contacts, or extra-granuloma multi-cell (CD11c-P25 T-cell) clusters came from 50 μm thick tissue sections. We scanned the entire depth above and below areas of interest by changing the focal length of the microscope to ensure that individual cell clusters were not 2 dimensional slices along the edge 3 dimensional granulomas.

### Adoptive Transfer of DsRed P25 T-cells

Leukocytes were collected from the spleen and lymph nodes (inguinal, mesenteric, renal, axillary, lumbar, and cervical) of DsRed-expressing, P25 transgenic mice^48^. 5 × 10^5^ P25 T-cells in 250 μL HBSS were then transferred intravenously into isoflurane-anesthetized mice by retro-orbital injection.

### Ex-vivo activation of P25 T-cells with sorted CD11c+ cells

Granuloma cells were isolated from wild-type mice 3 weeks after IP infection with DsRed BCG. Cells were stained with CD11c antibody and then sorted with the BD FACSJazz (BD Biosciences) into CD11c+ DsRed+ (infected) and CD11c+ DsRed- (uninfected) populations. Each sorted population was then cultured with leukocytes isolated from secondary lymph organs of P25 transgenic mice (20% of the total P25 cell population consists of VB11, mycobacterial-specific CD4 T-cells). The number of sorted CD11c+ cells added to P25 cultures was calculated to make up 8% of the total population. Positive control included P25 leukocytes with 1 ug/ml antigen 85b peptide, negative controls included P25 leukocytes alone. All cultures were incubated in 96 well plates in 250 μL cRPMI for 24 hours at 37 °C. Cells were then washed twice with FACS, stained with CD69 and VB11 antibodies, and then analyzed by flow cytometry.

### Granuloma Cell Migration

Granuloma dendritic cell migration was analyzed using the QCM 24-well invasion assay (Chemicon International). A total of 1.0 × 10^6^ granuloma cells harvested from 3 week DsRed BCG infected mice were placed in 0.1 ml of pre-warmed serum-free DMEM/Ham’s F12 medium and was added to the top of the transwells. 0.5 ml of medium supplemented with 240 ng/ml CCL21 (R&D Systems) or unsupplemented was added in the bottom chamber of the inserts. A further condition in which DsRed BCG (MOI: 1 BCG: 2 DC) was added to top of the transwells was also included. After 12 hours, granuloma cells were removed by Cell Detachment Solution from the upper and lower compartments. Migration index was calculated as a percentage of migrated granuloma cells relative to the initial amount of cells added to the top chamber. Following migration assay, cells were stained with CD11c (eBioscience) to assess the percentage of DsRed BCG-containing CD11c^high^ cells that migrated (bottom chamber) versus non-migrated (top chamber).

### Statistical Analysis

Results are expressed as means +/− s.e.m. Significance was determined using the students 2-tailed *t* test or one-way ANOVA. P < 0.05 was considered significant.

## Additional Information

**How to cite this article**: Harding, J. S. *et al.* Mycobacterium-Infected Dendritic Cells Disseminate Granulomatous Inflammation. *Sci. Rep.* **5**, 15248; doi: 10.1038/srep15248 (2015).

## Supplementary Material

Supplementary Information
