# Supplementary material for: Mycobacterium-Infected Dendritic Cells Disseminate Granulomatous Inflammation
Source: Sci Rep. 2015 Oct 30;5:15248. doi: 10.1038/srep15248 (PMC4626772; doi:10.1038/srep15248)
Supplement: Supplementary Information [file srep15248-s1.pdf]

Mycobacterium-Infected Dendritic Cells Disseminate Granulomatous Inflammation.

Supplementary Information

Jeffrey S. Harding[1,2], Aditya Rayasam[3], Heidi A. Schreiber[1,2], Zsuzsanna Fabry[1,2,3], Matyas Sandor[1,2]

[1]Department of Pathology and Laboratory Medicine, School of Medicine and Public Health, University of Wisconsin.

[2]Cellular and Molecular Pathology Training Program, University of Wisconsin Madison.

[3]Neuroscience Training Program, University of Wisconsin Madison

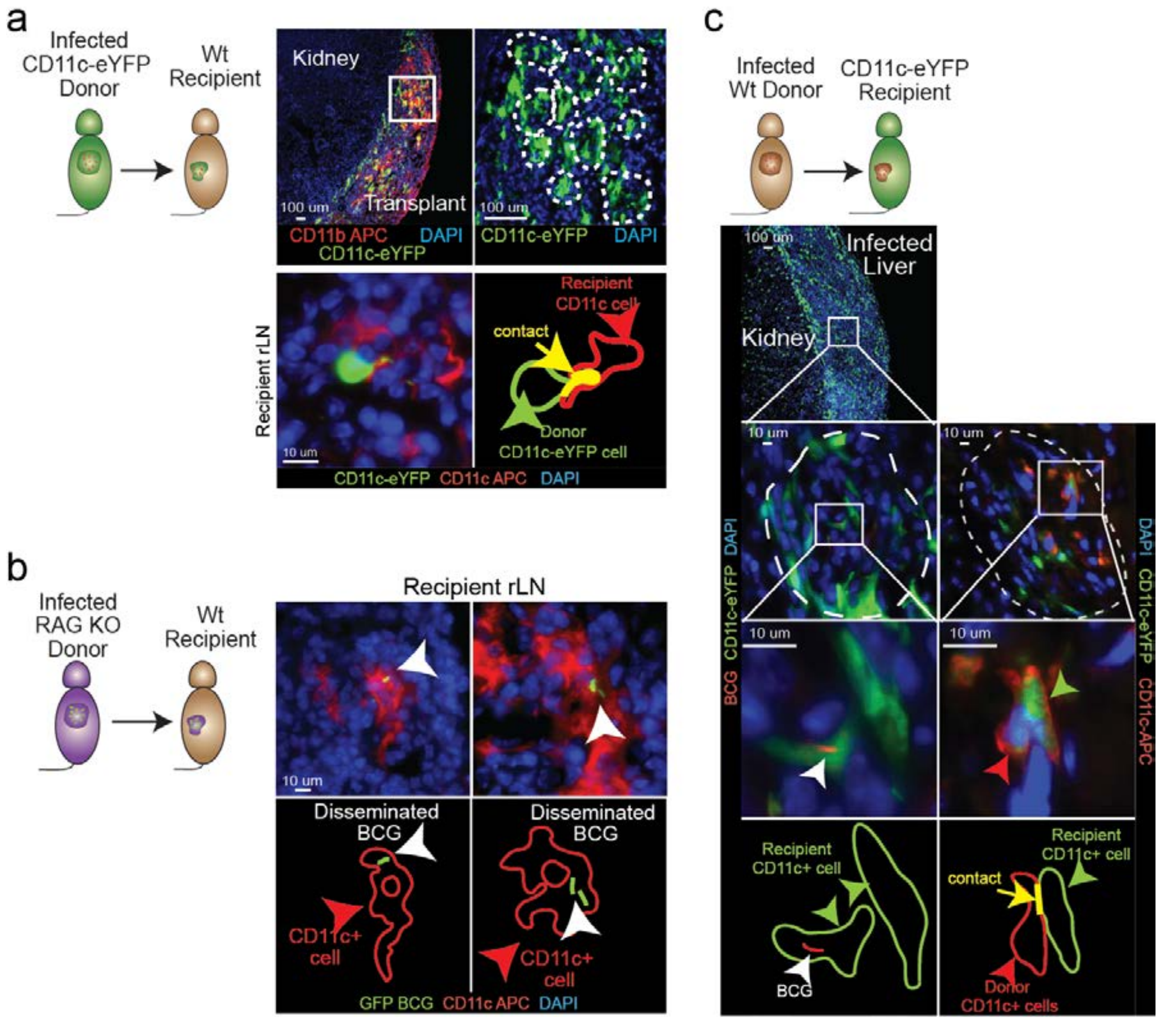

Supplemental Figure 1: Contact between peripheral and granuloma CD11c+ cells. **A.** 3wk-infected (IP with BCG) liver from CD11c-eYFP mice was transplanted underneath the kidney capsule of Wt recipients and left for 7 days. *Top row: m*icrographs of cross section of transplanted liver underneath the kidney capsule showing CD11b+ and CD11c+ cell-containing granulomas 7 days after transplant. Right images show magnification of representative area outlined in white. *Bottom Row: h*igh magnification micrograph of the renal lymph node (rLN) in Wt recipients after transplant of BCG-containing liver from CD11c-eYFP mice, showing contact between donor-derived CD11c-eYFP and recipient CD11c+ cell. **B.** Liver from RAG KO mice were acutely infected with GFP-BCG and transplanted into Wt recipients. Recipient rLN were harvested 7 days later. Red arrows indicate CD11c+ cells, white arrows show BCG. **C.** 3wk-infected (IP with BCG) liver from Wt mice was transplanted underneath the kidney capsule of CD11c-eYFP recipients and left for 7 days. *Top row:* Low magnification micrograph showing extensive infiltration of recipient CD11c-eYFP cells that migrated into the granuloma-containing liver. Individual granulomas (outlined by white dotted lines) that contain infiltrating CD11c-eYFP cells from the recipient (*second row, left and right)***,** can also be found infected BCG (*third and fourth row, left.* BCG indicated by white arrow*).* Co-staining with CD11c-APC antibody shows that some infiltrating CD11c-eYFP cells (green arrow in *third and fourth row, right)* make contact with donor CD11c-eYFP cells (red arrow in *third and fourth row, right).* Point of contact between donor CD11c cells (CD11c-APC antibody only) and recipient CD11c cells (colocalization with CD11c-APC antibody and CD11c-eYFP signal) shown with yellow arrow (fourth row, right).

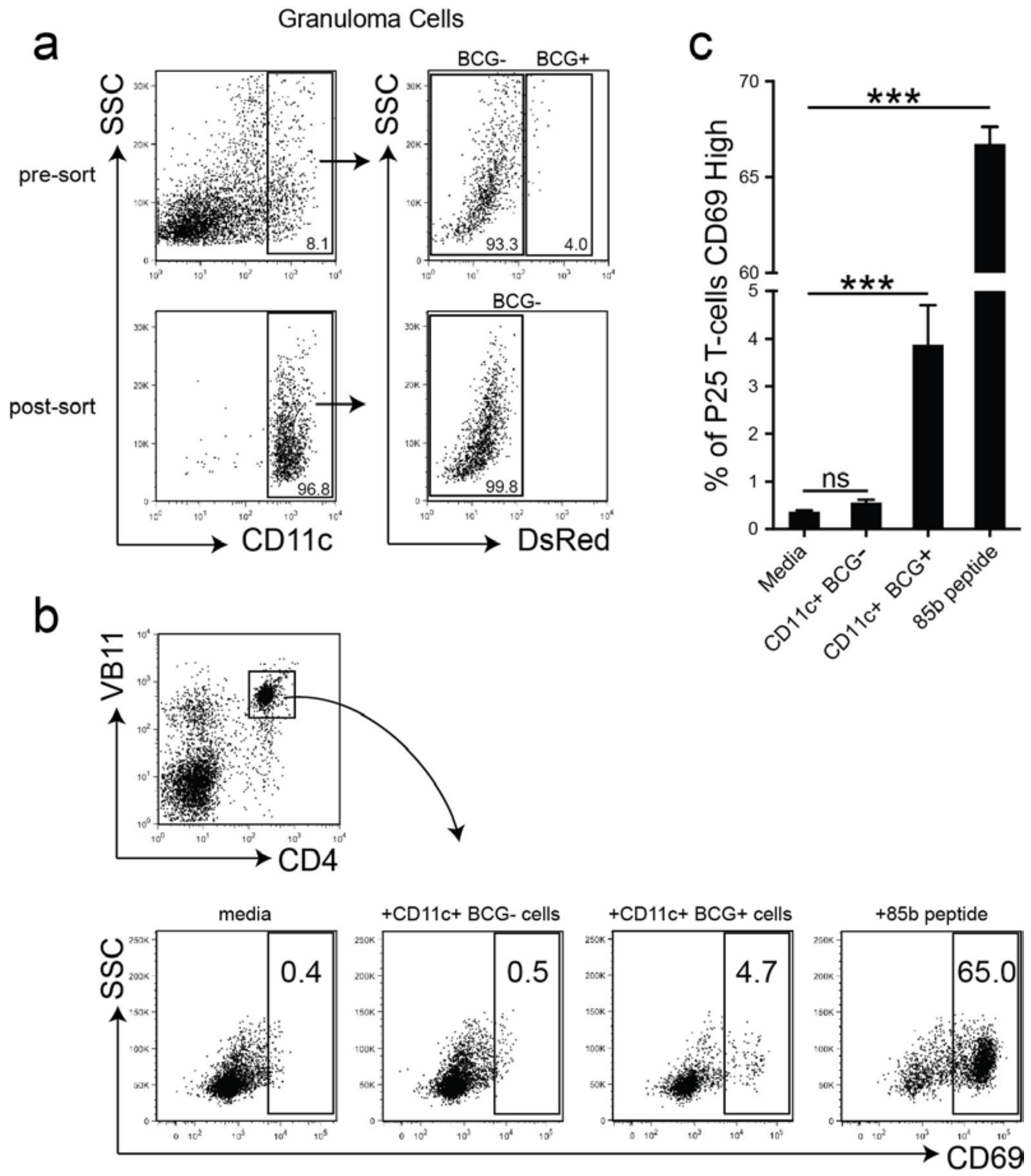

Supplemental Figure 2: Incubation of P25 T-cells with uninfected and infected CD11c+ granuloma cells. A. Granuloma cells from mice infected with DsRed BCG were isolated and stained with CD11c. Uninfected and infected CD11c+ cells can be identified based on colocalization with DsRed fluorescence (pre-sorted, top panels). Granuloma isolates were sorted by flow cytometry into CD11c^{high}BCG- and CD11c^{high}BCG+ populations (post-sort, bottom plots). Post-sort plot showing purified CD11c^{high}BCG+ cells is unavailable because all cells were needed for the proceeding assay (>99% purity of CD11c^{high}BCG+ was validated during sorting). **B.** Sorted CD11c^{high}BCG+ and CD11c^{high}BCG- cells from **A** were incubated with cultures containing P25 T-cells for 24 hours. Vβ11 vs. CD4 plot shows P25 T-cells from transgenic from mice identified in co-cultures with sorted CD11c^{high}cells. Activation of P25 T-cells in co-cultures was determined by CD69 expression. Controls included addition of media only (no CD11c^{high} cells), and addition of 1μg/ml 85b peptide only. **C.** Quantification of data from **B,** based on 3-5 replicate measurements. Error bars are mean +/- s.e.m**.** One-way ANOVA used to determine statistical significance. *P<0.05; **P<0.01; ***P<0.001.

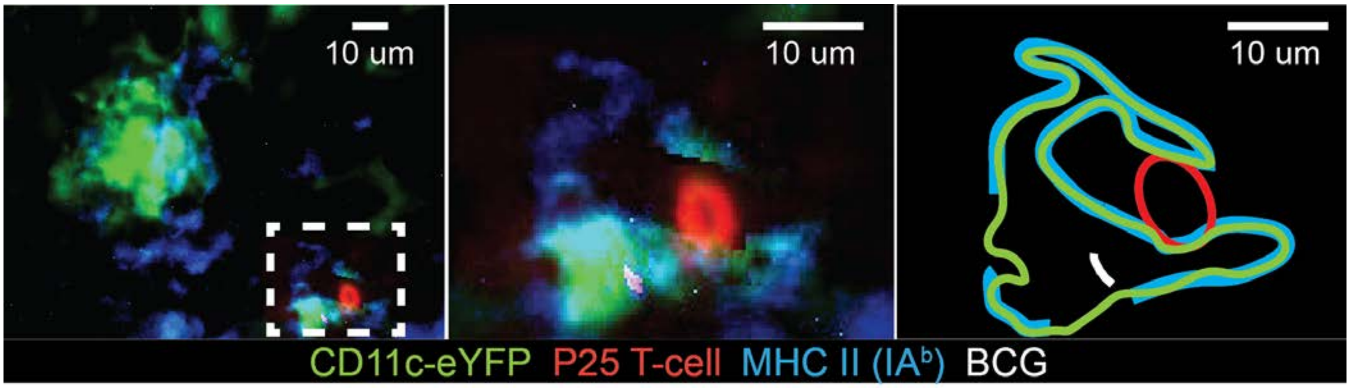

Supplemental Figure 2: BCG-containing DCs outside the granuloma in contact with P-25 T-cells are MHC II positive. Micrographs from 50um-thick mouse liver section 3 weeks after IP infection with $1x10^7$ CFU BCG. *Left image:* lower magnification area showing a mature/larger granuloma and a nearby extra-granuloma CD11c+ cell that contains BCG bacilli (white dotted box). The CD11c+ cell is in apparent contact with a mycobacterial-specific, DsRed-expressing P25 T-cell. *Middle Image:* Enlargement of area within white dotted box showing the colocalization of endogenous CD11c-eYFP and MHC II (IA^b) antibody fluorescent signal. *Right Image:* outlines showing the cell boundaries of CD11c+ and P25 T-cells, as well as BCG bacilli inside the CD11c+ cell.
